# Rush Medical College

**Published:** 1855-10

**Authors:** 


*Rush Medical College.*—The prospects of this Institution were never so flattering as at present. The new college building is just completed, and affords all the accommodations desired. The room for practical anatomy or dissections, is particularly capacious and pleasant. The time for opening this department and for commencing Clinical Instruction in the Hospital is fixed for the 1st Tuesday in October. There are already several students from a distance in attendance, visiting the wards of the Hospital daily and, waiting for the day appointed to commence the study of practical anatomy. In addition to the dissections and clinical instruction, two lectures a day on topics not fully embraced in the regular college term, will be given by the Faculty during the month of October; thus making the period of instruction in the college full five months to all who choose to attend.

We expect a full class, and trust that it will be both a pleasant and profitable winter to them.

